# Comparison of chlorhexidine based dressing versus simple occlusive dressing in preventing catheter related bloodstream infections at medical ICU in a resource constraint settings

**DOI:** 10.1186/2197-425X-3-S1-A812

**Published:** 2015-10-01

**Authors:** SMM Ali, SS Koul, MI Memon, TMU Pasha, S Afghan, F Tahir

**Affiliations:** Shaheed Zulfiqar Ali Bhutto Medical University Hospital, Islamabad, Pakistan

## Introduction

The cost of Catheter Related Bloodstream Infections (CRBSI) is substantial both in terms of morbidity and in terms of financial resources expended [1]. Rationale of current study was to evaluate two different dressing methods, chlorhexidine soaked gauze dressing and standard transparent polyurethane dressing in order to devise local guidelines for better infection control measures in our resource constraint settings.

## Objectives

To determine the efficacy of chlorhexidine soaked dressing on the CVC sites of patients admitted in critical care for the prevention of catheter tip colonization (CTC) and CRBSI.

## Methods

From the available data of medical ICU of Shaheed Zulfiqar Ali Bhutto medical university hospital, Islamabad fifty subjects were randomly selected who had chlorhexidine soaked gauze dressing over the CVC insertion site with a standard transparent polyurethane dressing over it (group I) and fifty were randomly selected who had standard transparent polyurethane CVC dressing applied directly to the CVC insertion site (group II). Blood cultures at day 3,6,9,12 and 15 were analyzed. CVC was removed and sent for colonization examination of all those patients who had shown clinical signs of infection around the catheter site at any time during their stay in ICU up till day 15, from all the remaining patients on day 15 and from all those patients who had died during this period or who had discharged before day 15.

## Results

Out of total 100 patients selected for the study, a total of 72 patients were BSI positive. Frequency of CTC was found to be 54 (54%) and frequency of CR-BSIs was 48 (48%) in both groups. In chlorhexidine group (group I) 42 % (n = 21) were CTC positive while the percentage was 66% (n = 33) in control group (group II) with RR of 0.617 (CI 0.413-0.921) for chlorhexidine group and OR of 0.373 (CI 0.166-0.839). P value was found to be 0.016 ( < 0.05). In chlorhexidine group (group I) 32 % (n = 16) were CR-BSIs positive while the percentage was 64% (n = 32) in control group (group II) with RR of 0.51(CI 0.326-0.797) for chlorhexidine group and OR of 0.265 (CI 0.166-0.606). P value was found to be 0.001 ( < 0.05).

## Conclusions

Chlorhexidine based dressings appeared to be an effective intervention to reduce rates of catheter tip colonization and catheter related blood stream infections.

## Grant Acknowledgment

No grant was taken for designing and execution for this study.
